# Emerging biomarkers for early detection of aplastic anemia in HIV: A narrative review

**DOI:** 10.1097/MD.0000000000047058

**Published:** 2026-01-16

**Authors:** Emmanuel Ifeanyi Obeagu, Getrude Uzoma Obeagu

**Affiliations:** a Department of Biomedical and Laboratory Science, Africa University, Mutare, Zimbabwe; b The Division of Molecular Medicine and Haematology, School of Pathology, Faculty of Health Sciences, University of the Witwatersrand, Johannesburg, South Africa; c School of Nursing Science, Kampala International University, Ishaka, Uganda.

**Keywords:** aplastic anemia, biomarkers, early detection, hematologic markers, HIV

## Abstract

Aplastic anemia (AA) in human immunodeficiency virus (HIV)-infected individuals is a complex condition that poses significant diagnostic and therapeutic challenges due to its overlap with HIV-related immune dysregulation. Early detection of AA is crucial for effective management and improved patient outcomes. Recent advances in biomarker research have identified several promising candidates that could facilitate earlier diagnosis and personalized treatment strategies. This review focuses on emerging biomarkers, including hematologic, immunologic, and molecular markers, which hold potential for the early detection of AA in HIV-infected patients. Hematologic biomarkers, such as reticulocyte count and bone marrow biopsy findings, provide initial insights into bone marrow dysfunction but may lack specificity. Immunologic biomarkers, including cytokine profiles and T-cell subsets, reflect the immune system’s role in AA pathogenesis and offer potential for earlier detection through assessment of inflammation and immune dysregulation. Additionally, molecular biomarkers, such as gene expression profiles and microRNAs, provide a deeper understanding of the underlying disease mechanisms and may enable more precise diagnosis and monitoring.

## 1. Introduction

Aplastic anemia (AA) is a severe hematologic condition characterized by the failure of bone marrow to produce adequate blood cells, leading to pancytopenia.^[1]^ In individuals with human immunodeficiency virus (HIV), AA presents additional complexities due to the interplay between HIV-induced immune dysregulation and the pathophysiology of AA.^[2,3]^ The early detection of AA in the context of HIV is critical for effective management and improving patient outcomes, as it allows for timely intervention and tailored treatment strategies.^[4]^ Recent advancements in biomarker research offer new opportunities for enhancing the early diagnosis and monitoring of AA in HIV-infected individuals. HIV infection can exacerbate the development of AA through several mechanisms.^[5]^ The virus induces chronic immune activation and inflammation, which can lead to the destruction of hematopoietic stem cell (HSCs) and impair bone marrow function. Additionally, HIV-associated immunosuppression may increase the risk of secondary causes of AA, such as infections or malignancies.^[6]^ The identification of reliable biomarkers for early detection of AA is essential, as traditional diagnostic methods may not detect the condition until it has progressed significantly. Biomarkers can provide valuable insights into the disease’s onset and progression, enabling earlier intervention and more precise management. Hematologic, immunologic, and molecular biomarkers each offer unique advantages for detecting AA and guiding treatment decisions.^[7]^ Hematologic biomarkers, such as reticulocyte count and bone marrow biopsy findings, are commonly used to assess bone marrow function and detect AA. However, these markers may lack specificity, particularly in the context of HIV-related changes in blood cell production. Advances in this area aim to improve the sensitivity and specificity of hematologic markers for early detection of AA.

Immunologic biomarkers offer additional insights into the role of the immune system in AA pathogenesis. Elevated levels of pro-inflammatory cytokines and alterations in T-cell subsets can reflect the immune dysregulation associated with AA in HIV-infected patients.^[8]^ These markers can help assess the inflammatory milieu and immune response, providing valuable information for early diagnosis and monitoring. Molecular biomarkers, including gene expression profiles and microRNAs, represent a promising area of research for AA detection. High-throughput technologies have enabled the identification of specific genetic and molecular signatures associated with AA, offering the potential for more precise and personalized diagnostic approaches. These biomarkers can provide a deeper understanding of disease mechanisms and guide targeted therapies. Integrating emerging biomarkers into clinical practice requires validation through extensive research and development of standardized assays. Collaboration between researchers, clinicians, and industry partners is crucial for translating biomarker discoveries into practical tools for early detection and management of AA. Effective implementation of these biomarkers can enhance diagnostic accuracy, improve treatment strategies, and ultimately lead to better patient outcomes.

### 1.1. Aim

The aim of this review is to explore and evaluate the emerging biomarkers for the early detection of AA in individuals living with HIV.

### 1.2. Justification

Early detection of AA in individuals living with HIV is critical, as delayed diagnosis can lead to severe complications, including life-threatening pancytopenia and bone marrow failure. Conventional diagnostic methods, such as bone marrow biopsies, are invasive, costly, and often performed late in the disease process, after significant clinical symptoms have appeared. This delays the initiation of appropriate therapies and reduces the chances of successful treatment. In the context of HIV, the risk of developing AA is heightened due to immune dysregulation, chronic inflammation, opportunistic infections, and the potential myelotoxic effects of antiretroviral therapy (ART). Given these factors, there is an urgent need for noninvasive, sensitive, and specific biomarkers that can detect AA at its early stages in HIV-infected individuals, facilitating timely intervention. Exploring emerging biomarkers offers a promising approach to improve diagnostic precision and early intervention in HIV-associated AA. Biomarkers, such as cytokines, telomere length, and exosomal microRNA (miRNA), may provide valuable insights into the disease’s progression and help reduce the morbidity and mortality associated with delayed diagnosis. This review is justified by the need to address the diagnostic gaps in AA among HIV patients and the potential of novel biomarkers to revolutionize early detection and clinical outcomes.

## 2. Materials and methods

This narrative review was conducted through a systematic literature search aimed at identifying recent advances in biomarkers for early detection of AA among individuals living with HIV. The primary databases searched included PubMed, Scopus, and Web of Science, covering publications from January 2000 to April 2025 to capture contemporary research trends. Search terms combined relevant keywords and Medical Subject Headings (MeSH) such as:

*“HIV,” “Human Immunodeficiency Virus,” “Aplastic Anemia,” “Bone Marrow Failure,” “Biomarkers,” “Hematologic Markers,” “Immunologic Markers,” “Molecular Markers,” “Early Detection,” and “Diagnosis.” Boolean operators* “AND” and “OR” were applied to refine the search.

Inclusion criteria were:

- Original research articles, reviews, and meta-analyses addressing biomarkers associated with AA in the context of HIV infection.
- Studies conducted in human subjects of any age group and geographical location.
- Publications in English.

Exclusion criteria were:

- Studies focusing solely on non-HIV-related AA.
- Case reports, editorials, and conference abstracts lacking full data.
- Animal or in vitro studies without clear clinical translational relevance.

After initial screening of titles and abstracts, full texts of relevant articles were retrieved for detailed evaluation. The quality of studies was appraised based on sample size, clarity of biomarker measurement methods, and relevance to early disease detection. Conflicting findings across studies were reconciled by comparative analysis and discussion among the authors, prioritizing data from larger cohorts and more recent investigations. Data extracted included biomarker types, methods of detection, associations with disease stages, and reported limitations. The information was synthesized thematically into hematologic, immunologic, and molecular biomarker categories to provide a structured narrative.

### 2.1. Ethical approval

Not applicable as this is a narrative review.

### 2.2. Pathophysiology of aplastic anemia in HIV

The pathophysiology of AA in HIV-infected individuals involves a complex interplay between HIV-induced immune dysregulation, bone marrow failure, and direct viral effects.^[3,9]^ HIV infection leads to chronic immune activation and dysregulation, which significantly impacts hematopoiesis.^[10]^ The virus primarily targets CD4+ T cells, causing their depletion and resulting in a compromised immune system.^[11]^ This immune dysfunction is associated with an increased inflammatory state, marked by elevated levels of pro-inflammatory cytokines such as tumor necrosis factor-alpha (TNF-α) and interleukin-6 (IL-6).^[12]^ These cytokines can contribute to bone marrow suppression and damage, exacerbating the development of AA.^[13]^ Chronic inflammation and immune activation disrupt the normal balance of hematopoiesis. Pro-inflammatory cytokines can inhibit the proliferation and differentiation of HSCs and progenitor cells, leading to reduced production of blood cells.^[14]^ Additionally, elevated cytokine levels can induce apoptosis in hematopoietic cells, further compromising bone marrow function and contributing to the onset of AA.

HIV can have direct effects on the bone marrow, impacting its ability to produce blood cells.^[15]^ The virus may infect bone marrow cells, including HSCs, either directly or through interactions with viral proteins. This direct infection can impair the functionality of HSCs and progenitor cells, leading to decreased hematopoiesis and increased risk of developing AA. In addition to direct infection, HIV-related changes in bone marrow microenvironment can disrupt normal hematopoiesis.^[16,17]^ HIV-induced alterations in bone marrow stromal cells, which provide support and regulatory signals for hematopoietic cells, can further impair blood cell production. These changes may contribute to the development of AA by creating an inhospitable environment for hematopoiesis. In HIV-infected individuals, the immune system may also play a role in causing bone marrow damage through autoimmune mechanisms.^[18]^ The chronic immune activation associated with HIV can lead to the production of autoantibodies against hematopoietic cells or bone marrow components.^[19]^ This autoimmune response can result in the destruction of hematopoietic cells and contribute to the development of AA. Furthermore, the presence of opportunistic infections and other complications in HIV-infected individuals can exacerbate immune-mediated damage to the bone marrow.^[20]^ For example, infections with certain pathogens may trigger immune responses that inadvertently target bone marrow cells, worsening the anemia and complicating the management of AA. Genetic and epigenetic factors may also influence the susceptibility to AA in the context of HIV.^[21]^ Genetic predispositions affecting immune function and hematopoiesis can interact with HIV-induced changes, increasing the risk of developing AA.^[22,23]^ For instance, genetic variations in cytokine genes or genes involved in DNA repair and hematopoiesis may modulate the severity of AA in HIV-infected individuals. Epigenetic modifications, such as changes in deoxyribonucleic acid (DNA) methylation and histone acetylation, can affect gene expression related to immune response and hematopoiesis. These modifications may be influenced by chronic HIV infection and contribute to the pathogenesis of AA. The pathophysiology of AA in HIV-infected individuals underscores the need for a comprehensive approach to diagnosis and management.^[24]^ Recognizing the multifactorial nature of AA in the context of HIV is essential for identifying effective biomarkers and developing targeted therapies. Addressing both the immune dysregulation and direct viral effects on bone marrow is crucial for improving patient outcomes and optimizing treatment strategies.

### 2.3. Emerging hematologic biomarkers

The identification and utilization of emerging hematologic biomarkers offer significant potential for the early detection, diagnosis, and management of AA in HIV-infected individuals.^[25]^ These biomarkers provide insights into the underlying pathophysiology of AA and help distinguish it from other conditions that may present similarly in the context of HIV. This section explores several emerging hematologic biomarkers, highlighting their roles and potential clinical applications.

#### 2.3.1. Reticulocyte count

Reticulocytes are immature red blood cells released into the bloodstream from the bone marrow. The reticulocyte count is a key indicator of bone marrow activity and erythropoiesis. In the context of AA, reticulocyte levels are typically low due to impaired red blood cell production. While a low reticulocyte count alone is not specific to AA, it can provide early clues about bone marrow dysfunction. Monitoring reticulocyte counts in HIV-infected patients can be useful for detecting early signs of anemia and assessing the bone marrow’s response to anemia.^[26]^ Low reticulocyte counts in HIV-infected individuals may prompt further evaluation to determine the underlying cause of bone marrow failure.^[27]^ Although reticulocyte count is a useful initial marker, it should be interpreted in conjunction with other clinical findings and diagnostic tests.

#### 2.3.2. Bone marrow biopsy findings

Bone marrow biopsy remains a critical diagnostic tool for evaluating bone marrow function and diagnosing AA. Advances in imaging techniques and histopathological analysis have improved the ability to detect early changes associated with AA. Key findings in bone marrow biopsies for AA include decreased cellularity, an increase in fat spaces, and the presence of dysplastic changes. These findings help confirm the diagnosis of AA and differentiate it from other hematologic disorders. Bone marrow biopsy provides definitive information about bone marrow morphology and cellularity, which is crucial for diagnosing AA. In HIV-infected patients, bone marrow biopsy can help identify any coexisting conditions that might contribute to bone marrow failure.^[28]^

#### 2.3.3. Hemoglobin F (fetal hemoglobin) levels

Hemoglobin F (HbF) is the fetal form of hemoglobin that persists at low levels in adults. Elevated levels of HbF can be associated with certain hematologic conditions, including AA. In some cases, increased HbF levels may be observed as a compensatory response to anemia or bone marrow dysfunction. Assessing HbF levels can provide additional information about the nature and severity of anemia in HIV-infected individuals. Monitoring HbF levels can offer insights into the underlying pathology of anemia in HIV-infected patients and aid in distinguishing AA from other causes of anemia.^[29]^ Elevated HbF levels should be interpreted alongside other clinical and diagnostic findings.

#### 2.3.4. Erythropoietin levels

Erythropoietin (EPO) is a hormone produced by the kidneys that stimulates red blood cell production. In cases of AA, EPO levels may be elevated as the body attempts to compensate for low red blood cell counts. However, in the context of AA associated with HIV, the relationship between EPO levels and bone marrow function can be complex. Evaluating EPO levels can provide additional context for understanding anemia and guiding treatment. Elevated EPO levels in HIV-infected individuals with anemia may suggest a compensatory response to low red blood cell counts.^[30]^ EPO levels should be assessed alongside other markers to provide a comprehensive evaluation of anemia.

#### 2.3.5. Soluble CD26 and CD27

Soluble CD26 (sCD26) and soluble CD27 (sCD27) are immune markers that reflect T-cell activation and immune system dysregulation. Elevated levels of sCD26 and sCD27 have been associated with chronic inflammation and immune activation, both of which can contribute to bone marrow dysfunction in HIV-infected individuals.^[31]^ These markers may provide insights into the immune-mediated aspects of AA. Measuring sCD26 and sCD27 levels can help assess the degree of immune activation and inflammation in HIV-infected patients with AA. These markers can complement other diagnostic tests and provide a better understanding of the disease’s immunological components.

#### 2.3.6. Anemia-related microRNAs

miRNAs are small, noncoding RNAs that regulate gene expression and have been implicated in various hematologic disorders. Specific miRNAs, such as miR-146a and miR-155, have shown potential as biomarkers for anemia due to their roles in regulating inflammation and hematopoiesis. Profiling miRNAs in patients with AA can offer insights into the molecular mechanisms underlying the disease. miRNAs associated with anemia can serve as potential biomarkers for AA in HIV-infected individuals.^[5]^ Their profiles can provide valuable information about disease mechanisms and guide treatment strategies.

#### 2.3.7. CD34+ stem cell counts

CD34+ cells are HSCs found in the bone marrow and peripheral blood. These cells are crucial for hematopoiesis and their counts can provide insights into bone marrow function. In AA, the number of CD34+ cells may be reduced due to impaired hematopoiesis. Monitoring CD34+ cell counts can help evaluate the extent of bone marrow failure and the potential for recovery. Assessing CD34+ cell counts can aid in diagnosing AA and monitoring treatment response. A low CD34+ count may indicate severe bone marrow dysfunction and the need for further therapeutic interventions.^[32]^

#### 2.3.8. Growth factor levels

Growth factors such as granulocyte-colony stimulating factor and thrombopoietin play crucial roles in regulating hematopoiesis. Alterations in the levels of these growth factors can reflect the bone marrow’s response to AA and provide insights into disease mechanisms. Elevated or decreased levels of growth factors can indicate underlying issues with hematopoiesis in HIV-infected patients.^[33]^ Measuring growth factor levels can offer additional information about the status of bone marrow function and help guide therapeutic decisions. Abnormal growth factor levels should be interpreted in the context of other diagnostic findings.

### 2.4. Emerging biomarkers for early detection of aplastic anemia in HIV

The identification of early biomarkers for AA in HIV-infected individuals holds considerable promise for improving diagnostic accuracy and guiding therapeutic decisions. These biomarkers, which reflect various aspects of bone marrow function and immune dysregulation, can be broadly categorized into hematologic, immunologic, and molecular indicators. This structured approach provides a clearer understanding of the pathophysiological processes underlying AA in HIV and offers a framework for clinical application.^[25,26]^

### 2.5. Hematologic biomarkers

Hematologic parameters remain foundational to the diagnosis of bone marrow failure syndromes. In the context of HIV-associated AA, early hematologic changes may precede overt pancytopenia and serve as important warning signals. A key marker is the absolute reticulocyte count, which is typically low in AA due to impaired erythropoiesis. Additionally, the peripheral CD34+ HSC count – reflecting the circulating pool of progenitor cells – may be significantly reduced before substantial marrow hypocellularity becomes evident. Another useful indicator is the red cell distribution width, which may increase in the early stages of ineffective erythropoiesis. Mean corpuscular volume changes, especially macrocytosis in the absence of vitamin B_12_ or folate deficiency, may also suggest early marrow stress or toxicity, particularly in patients receiving zidovudine-based ART. Monitoring these hematologic markers can aid in recognizing subtle deviations from normal marrow activity before clinical symptoms of AA become pronounced.^[5,27–33]^

### 2.6. Immunologic biomarkers

Immune-mediated destruction of hematopoietic progenitor cells is a hallmark of acquired AA, and in HIV, persistent immune activation exacerbates this process. Several cytokines, including interferon-γ and TNF-α, have been implicated in marrow suppression. Elevated levels of these cytokines promote apoptosis and inhibit the proliferation of HSCs. Interleukin-6 (IL-6) and interleukin-1 beta (IL-1β) also contribute to the inflammatory milieu that impairs hematopoiesis in HIV-infected individuals.^[34–38]^ Furthermore, soluble immune checkpoint molecules, such as programmed death-1 and cytotoxic T-lymphocyte-associated protein 4, have been observed at elevated levels in both AA and advanced HIV. These molecules may reflect ongoing immune exhaustion and bone marrow immune dysregulation. Markers such as Fas (CD95) and Fas ligand, which are involved in apoptotic signaling, are upregulated in T cells and bone marrow progenitors during immune-mediated marrow injury. Their detection in plasma or bone marrow aspirates may provide early evidence of immune-mediated cytopenia and incipient AA.^[39–41]^

### 2.7. Molecular biomarkers

At the molecular level, several promising biomarkers have emerged with potential relevance for the early detection of AA in HIV. miRNAs – small, noncoding RNAs that regulate gene expression – have been implicated in hematopoietic regulation. Aberrant expression of miR-150, miR-155, and miR-29b has been associated with impaired stem cell function and could serve as early, minimally invasive markers for marrow dysfunction. Telomere length, a surrogate for stem cell replicative capacity, has been found to be significantly shortened in patients with idiopathic and immune-mediated AA.^[42–44]^ HIV infection may accelerate telomere erosion due to chronic immune activation and oxidative stress. Measurement of telomere attrition in leukocytes may, therefore, help identify HIV-positive individuals at risk for AA before bone marrow failure is clinically apparent.^[45–47]^ Other markers of oxidative stress – such as malondialdehyde and glutathione levels – have been correlated with increased cellular damage in the marrow microenvironment. Additionally, markers like gamma H2A histone family member X, indicative of DNA double-strand breaks, provide evidence of genomic instability in hematopoietic cells and may signify early stages of marrow exhaustion.^[48]^ Advances in proteomics have also identified novel plasma proteins involved in the maintenance of marrow niche homeostasis, such as osteopontin, stromal cell-derived factor-1, and vascular cell adhesion molecule-1. These molecules play roles in stem cell retention, migration, and survival, and their dysregulation may signal impending marrow failure (Tables 1 and 2).^[49]^

**Table 1:** Biomarkers for early detection of aplastic anemia in HIV.

Biomarker	Mechanism of action	Type	Sensitivity	Specificity	Diagnostic value
Thrombopoietin	Stimulates platelet production and reflects bone marrow activity	Glycoprotein	High	Moderate	Early marker for bone marrow failure in HIV-infected individuals
CD34 + Cells	Indicator of hematopoietic stem cell activity	Surface marker	High	High	Predicts early bone marrow dysfunction
Telomere length	Measures chromosomal stability and cell aging	Genetic marker	Moderate	High	Associated with bone marrow exhaustion and early AA detection
Exosomal miRNAs	Regulate gene expression and immune response	miRNA biomarker	High	High	Novel noninvasive marker for bone marrow failure
Cytokine profiles	Reflect immune activation and inflammation	Protein marker	Moderate	Moderate	Early indicator of immune dysregulation leading to AA

AA = aplastic anemia, HIV = human immunodeficiency virus, miRNA = microRNA.

**Table 2:** Categories of biomarkers for aplastic anemia in HIV.

Category	Examples of biomarkers	Role in detection of AA
Immune activation markers	Cytokines (IL-6, IL-10)	Early signs of immune dysregulation and bone marrow stress
Hematopoietic stem cell markers	CD34+, CD133+ cells	Reflects stem cell function and early bone marrow depletion
Genetic markers	Telomere length, gene mutations	Predicts chromosomal instability and premature bone marrow failure
Exosomal biomarkers	miR-122, miR-155	Involved in regulating immune response and cell death in AA
Cytokine and inflammatory markers	IFN-γ, TNF-α	Reflect chronic immune activation and inflammation in HIV-associated AA

AA = aplastic anemia, HIV = human immunodeficiency virus, IFN-γ = interferon gamma, IL = interleukin, TNF-α = tumor necrosis factor alpha.

### 2.8. Diagnostic and therapeutic implications

Emerging molecular biomarkers provide significant diagnostic and therapeutic advantages for managing AA in HIV-infected individuals. By offering insights into disease mechanisms, progression, and response to treatment, these biomarkers can enhance clinical decision-making and lead to more personalized treatment strategies.

#### 2.8.1. Diagnostic implications

##### 2.8.1.1. Early detection and diagnosis

Molecular biomarkers such as genetic variants, DNA methylation patterns, and microRNAs can significantly enhance the early detection and diagnosis of AA.^[50]^ For instance, identifying specific genetic mutations (e.g., in TP53 or DKC1) associated with bone marrow failure can aid in diagnosing AA and distinguishing it from other hematologic disorders. Similarly, abnormal DNA methylation patterns can indicate epigenetic alterations linked to AA, providing additional diagnostic clues.

##### 2.8.1.2. Risk stratification

Profiling molecular biomarkers allows for more precise risk stratification of patients.^[51]^ For example, high levels of pro-inflammatory cytokines or altered T-cell subsets can indicate severe immune dysregulation and a higher risk of progressive AA. Genetic and epigenetic profiles can also predict disease severity and progression, enabling clinicians to tailor surveillance and intervention strategies based on individual risk profiles.

##### 2.8.1.3. Monitoring disease progression

Biomarkers such as circulating tumor DNA (ctDNA) and proteomic markers offer noninvasive methods to monitor disease progression and treatment response.^[52]^ Regular monitoring of ctDNA levels can provide insights into changes in bone marrow cell turnover and disease dynamics. Proteomic analyses can reveal alterations in protein expression linked to disease progression and response to therapy, allowing for real-time assessment of treatment efficacy.

##### 2.8.1.4. Differentiation from other disorders

Molecular biomarkers can help differentiate AA from other conditions with similar clinical presentations. For instance, autoantibody profiles and specific genetic mutations can distinguish AA from other forms of anemia or bone marrow disorders. Accurate diagnosis is crucial for appropriate treatment planning and management.

#### 2.8.2. Therapeutic implications

##### 2.8.2.1. Personalized treatment strategies

The use of molecular biomarkers enables the development of personalized treatment strategies. For instance, identifying specific genetic mutations or epigenetic alterations can guide targeted therapies aimed at correcting underlying abnormalities. Personalized approaches can improve treatment outcomes and reduce the risk of adverse effects by tailoring interventions to individual patient profiles.

##### 2.8.2.2. Targeted therapeutic interventions

Emerging molecular biomarkers can inform the selection of targeted therapeutic interventions. For example, identifying overexpression of immune checkpoint molecules like programmed death-1 or PD-L1 can suggest the use of immune checkpoint inhibitors to modulate immune responses and improve hematopoiesis. Similarly, targeted therapies based on specific genetic or epigenetic abnormalities can address the root causes of AA and enhance treatment efficacy.

##### 2.8.2.3. Monitoring treatment response

Molecular biomarkers play a crucial role in monitoring treatment response and adjusting therapeutic strategies. Regular assessment of biomarkers such as miRNAs, ctDNA, and proteomic profiles can provide real-time feedback on treatment efficacy and guide adjustments to therapy. This dynamic approach ensures that treatment remains effective and responsive to changes in disease status.^[52]^

##### 2.8.2.4. Management of complications

Biomarkers can also help manage complications associated with AA and its treatment. For example, monitoring levels of pro-inflammatory cytokines and immune activation markers can guide the management of inflammation-related complications and adjust anti-inflammatory treatments as needed. Identifying biomarkers linked to treatment-induced toxicity can also help mitigate adverse effects and improve patient safety.^[53]^

##### 2.8.2.5. Prognostic indicators

Molecular biomarkers serve as valuable prognostic indicators, helping to predict disease outcomes and long-term prognosis. Genetic and epigenetic profiles, along with levels of specific biomarkers, can provide insights into the likely course of the disease and response to treatment. This information is essential for guiding long-term management strategies and setting realistic expectations for patient outcomes.^[53]^

### 2.9. Challenges and research gaps

Despite significant advances in identifying potential biomarkers for early detection of AA in HIV-infected individuals, several challenges remain that limit their clinical utility and broader application. One of the foremost limitations concerns the specificity of these biomarkers. Many hematologic and immunologic indicators associated with AA overlap with changes induced by HIV infection itself or by other common HIV-related complications such as opportunistic infections and drug toxicities. For example, elevated pro-inflammatory cytokines like TNF-α and interferon-γ are not exclusive to AA but are also characteristic of generalized HIV-driven immune activation. This lack of specificity complicates the interpretation of biomarker elevations and reduces their reliability in distinguishing early AA from other causes of cytopenia in HIV.^[48]^ Another significant challenge is the variability of biomarker expression across diverse HIV-infected populations. Differences in genetic background, HIV subtype, coinfections (e.g., tuberculosis, hepatitis), and nutritional status can influence baseline biomarker levels and disease progression patterns. Such heterogeneity makes it difficult to establish universal cutoff values or diagnostic thresholds applicable across regions and patient cohorts, particularly in resource-limited settings where the burden of HIV is greatest.^[49]^

Moreover, many biomarkers demonstrate temporal fluctuations during the natural history of HIV-associated bone marrow failure. Biomarker levels may vary according to the stage of AA development – from initial marrow stress and progenitor cell depletion to advanced hypocellularity and pancytopenia. These dynamic changes require longitudinal monitoring to accurately capture early disease signals, yet most studies to date have been cross-sectional. The absence of standardized timelines for biomarker measurement hinders early diagnosis and timely intervention.^[50,51]^ Additionally, comorbid infections and ART regimens pose further complications in biomarker interpretation. Opportunistic infections can independently alter hematologic and immune parameters, while certain ART drugs, such as zidovudine and ganciclovir, are known to cause marrow suppression, confounding the clinical picture. Distinguishing AA driven by HIV-mediated immune dysregulation from drug-induced cytopenias or infection-related marrow impairment remains a critical clinical challenge.^[52]^ Most current studies are limited by small sample sizes, lack of longitudinal data, and inconsistent methodological approaches, underscoring the need for well-designed, multicenter prospective studies. There is also a need to integrate biomarker research with functional assays and clinical parameters to develop composite predictive models with enhanced diagnostic accuracy.^[53]^

## 3. Conclusion

the early detection of AA in individuals living with HIV is a critical challenge that requires innovative diagnostic approaches. Traditional methods, while effective, often diagnose the condition too late, leading to poor patient outcomes. The exploration of emerging biomarkers such as thrombopoietin, hematopoietic stem cell markers, immune activation markers, cytokine profiles, and exosomal miRNAs offers significant promise in addressing this diagnostic gap. These biomarkers reflect the underlying pathophysiology of AA and provide noninvasive options that could be incorporated into routine monitoring protocols for high-risk HIV patients. The interaction between HIV and the immune system contributes significantly to the development of AA, as chronic immune activation, direct viral effects, and antiretroviral drug toxicity place additional stress on the bone marrow. Biomarkers that capture these interactions, such as immune activation markers and cytokine profiles, provide valuable insights into the early stages of bone marrow dysfunction. Early detection allows for timely intervention, which is crucial in preventing the progression of AA and improving patient survival. Moreover, the molecular and cellular markers identified in this review, such as CD34+ cells and telomere length, hold significant potential for understanding the broader implications of bone marrow failure in the context of HIV. These biomarkers not only enable early diagnosis but also offer a deeper understanding of the mechanisms driving AA. Such insights are essential for the development of targeted therapies that could improve both hematological and immune recovery in HIV patients.

## Author contributions

**Conceptualization:** Emmanuel Ifeanyi Obeagu.

**Methodology:** Emmanuel Ifeanyi Obeagu, Getrude Uzoma Obeagu.

**Resources:** Emmanuel Ifeanyi Obeagu.

**Validation:** Emmanuel Ifeanyi Obeagu, Getrude Uzoma Obeagu.

**Visualization:** Emmanuel Ifeanyi Obeagu, Getrude Uzoma Obeagu.

**Writing – original draft:** Emmanuel Ifeanyi Obeagu, Getrude Uzoma Obeagu.

**Writing – review & editing:** Emmanuel Ifeanyi Obeagu, Getrude Uzoma Obeagu.

## References

[1] Miano M Dufour C. The diagnosis and treatment of aplastic anemia: a review. Int J Hematol. 2015;101:527–35. 25837779 10.1007/s12185-015-1787-z

[2] Obeagu EI Kanu SN. Role of immune dysregulation in aplastic anemia pathogenesis in HIV. Elite J Health Sci. 2024;2:112–28.

[3] Obeagu EI. Immunological insights into aplastic anemia within the context of HIV: unraveling the complex interplay. Elite J Health Sci. 2023;1:14–24.

[4] Ndlovu Z Burton R Stewart R. Framework for the implementation of advanced HIV disease diagnostics in sub-Saharan Africa: programmatic perspectives. The Lancet HIV. 2020;7:e514–20. 32473102 10.1016/S2352-3018(20)30101-6

[5] Bandera A Taramasso L Bozzi G. HIV-associated neurocognitive impairment in the modern ART era: are we close to discovering reliable biomarkers in the setting of virological suppression? Front Aging Neurosci. 2019;11:187. 31427955 10.3389/fnagi.2019.00187 PMC6687760

[6] Ji Y Lu H. Malignancies in HIV-Infected and AIDS patients. Adv Exp Med Biol. 2017;1018:167–79. 29052137 10.1007/978-981-10-5765-6_10

[7] Wlodarski MW Gondek LP Nearman ZP. Molecular strategies for detection and quantitation of clonal cytotoxic T-cell responses in aplastic anemia and myelodysplastic syndrome. Blood. 2006;108:2632–41. 16614248 10.1182/blood-2005-09-3902 PMC1895579

[8] Borges-Almeida E Milanez HM Vilela MM. The impact of maternal HIV infection on cord blood lymphocyte subsets and cytokine profile in exposed non-infected newborns. BMC Infect Dis. 2011;11:1. 21291536 10.1186/1471-2334-11-38 PMC3040712

[9] Obeagu EI Akinleye CA. A review on the clinical manifestations and complications of aplastic anemia in HIV. Elite J Public Health. 2024;2:18–30.

[10] Obeagu EI Iheanacho MC. Aplastic anemia in HIV: role of hematopoietic cytokines and growth factors. Elite J Lab Med. 2024;2:42–56.

[11] Swain SL McKinstry KK Strutt TM. Expanding roles for CD4+ T cells in immunity to viruses. Nat Rev Immunol. 2012;12:136–48. 22266691 10.1038/nri3152 PMC3764486

[12] Kany S Vollrath JT Relja B. Cytokines in inflammatory disease. Int J Mol Sci . 2019;20:6008. 31795299 10.3390/ijms20236008 PMC6929211

[13] Li JP Zheng CL Han ZC. Abnormal immunity and stem/progenitor cells in acquired aplastic anemia. Crit Rev Oncol Hematol. 2010;75:79–93. 20045349 10.1016/j.critrevonc.2009.12.001

[14] Jahandideh B Derakhshani M Abbaszadeh H. The pro-Inflammatory cytokines effects on mobilization, self-renewal and differentiation of hematopoietic stem cells. Hum Immunol. 2020;81:206–17. 32139091 10.1016/j.humimm.2020.01.004

[15] Alexaki A Wigdahl B. HIV-1 infection of bone marrow hematopoietic progenitor cells and their role in trafficking and viral dissemination. PLoS Pathog. 2008;4:e1000215. 19112504 10.1371/journal.ppat.1000215 PMC2603331

[16] Koka PS Reddy ST. Cytopenias in HIV infection: mechanisms and alleviation of hematopoietic inhibition. Curr HIV Res. 2004;2:275–82. 15279591 10.2174/1570162043351282

[17] Obeagu EI. Aplastic anemia and HIV: the role of inflammatory cytokines. Elite J Lab Med. 2024;2:62–77.

[18] Chauvin M Sauce D. Mechanisms of immune aging in HIV. Clin Sci (Colch). 2022;136:61–80. 34985109 10.1042/CS20210344

[19] Pascutti MF Erkelens MN Nolte MA. Impact of viral infections on hematopoiesis: from beneficial to detrimental effects on bone marrow output. Front Immunol. 2016;7:364. 27695457 10.3389/fimmu.2016.00364 PMC5025449

[20] Bhowmik A Banerjee P. Hematological manifestation in HIV infected children. J Coll Physicians Surg Pak. 2015;25:119–23. 25703756

[21] Sundermann EE Hussain MA Moore DJ; HNRP Group. Inflammation-related genes are associated with epigenetic aging in HIV. J Neurovirol. 2019;25:853–65. 31286441 10.1007/s13365-019-00777-4 PMC6923602

[22] Obeagu EI Kanu SN. Understanding the Genetic Susceptibility to Aplastic Anemia in HIV. Elite J Lab Med. 2024;2:1–6.

[23] Obeagu EI Iheanacho MC. Genetic factors influencing aplastic anemia in the context of HIV. Elite J Health Sci. 2024;2:143–55.

[24] Feinstein MJ Hsue PY Benjamin LA; American Heart Association Prevention Science Committee of the Council on Epidemiology and Prevention and Council on Cardiovascular and Stroke Nursing; Council on Clinical Cardiology; and Stroke Council. Characteristics, prevention, and management of cardiovascular disease in people living with HIV: a scientific statement from the American Heart Association. Circulation. 2019;140:e98–e124. 31154814 10.1161/CIR.0000000000000695 PMC7993364

[25] You X Yang Q Yan K. Multi-omics profiling identifies pathways associated with CD8+ T-cell activation in severe aplastic anemia. Front Genet. 2022;12:790990. 35058969 10.3389/fgene.2021.790990 PMC8764265

[26] Omosigho PO Ilesanmi AO Asonye LN. Gender disparity in the reticulocyte count of HIV-positive patients on HAART management in Ilorin. J Clin Exp Hematol. 2024;3:22–8.

[27] Marchionatti A Parisi MM. Anemia and thrombocytopenia in people living with HIV/AIDS: a narrative literature review. Int Health. 2021;13:98–109. 32623456 10.1093/inthealth/ihaa036 PMC7902680

[28] Chetty C Musekwa E Chapanduka ZC. The value of bone marrow examinations performed in the investigation of HIV infected patients with cytopenias. Int J Lab Hematol. 2023;45:553–61. 37129086 10.1111/ijlh.14079

[29] Kontoghiorghes GJ. Drug selection and posology, optimal therapies and risk/benefit assessment in medicine: the paradigm of iron-chelating drugs. Int J Mol Sci. 2023;24:16749. 38069073 10.3390/ijms242316749 PMC10706143

[30] Redig AJ Berliner N. Pathogenesis and clinical implications of HIV-related anemia in 2013. Hematology Am Soc Hematol Educ Program. 2013;2013:377–81. 24319207 10.1182/asheducation-2013.1.377

[31] Lichtfuss GF Hoy J Rajasuriar R Kramski M Crowe SM Lewin SR. Biomarkers of immune dysfunction following combination antiretroviral therapy for HIV infection. Biomarkers Med. 2011;5:171–86. 10.2217/bmm.11.15 21473720

[32] Maciejewski JP Selleri C Sato T Anderson S Young NS. Increased expression of Fas antigen on bone marrow CD34+ cells of patients with aplastic anaemia. Br J Haematol. 1995;91:245–52. 7577642 10.1111/j.1365-2141.1995.tb05277.x

[33] Scadden DT. Hematologic disorders and growth factor support in HIV infection. Hematol Oncol Clin North Am. 1996;10:1149–61. 8880202 10.1016/s0889-8588(05)70390-7

[34] Buys H Muloiwa R Bamford C Eley B. Klebsiella pneumoniae bloodstream infections at a South African children’s hospital 2006-2011, a cross-sectional study. BMC Infect Dis. 2016;16:570. 27751185 10.1186/s12879-016-1919-y PMC5067886

[35] McInally S Wall K Yu T. Elevated levels of inflammatory plasma biomarkers are associated with risk of HIV infection. Retrovirology. 2021;18:1–1. 33731158 10.1186/s12977-021-00552-6 PMC7968240

[36] Gonzalez SM Taborda NA Rugeles MT. Role of different subpopulations of CD8+ T cells during HIV exposure and infection. Front Immunol. 2017;8:936. 28824656 10.3389/fimmu.2017.00936 PMC5545716

[37] García‐Calvo X Bolao F Sanvisens A. Significance of markers of monocyte activation (CD163 and sCD14) and inflammation (IL‐6) in patients admitted for alcohol use disorder treatment. Alcohol Clin Exp Res. 2020;44:152–8. 31797394 10.1111/acer.14228

[38] Abbas M Steffens S Bellut M. Do programmed death 1 (PD-1) and its ligand (PD-L1) play a role in patients with non-clear cell renal cell carcinoma? Med Oncol. 2016;33:1–7. 27165272 10.1007/s12032-016-0770-8

[39] Ouyang J Yan J Zhou X. Relevance of biomarkers indicating gut damage and microbial translocation in people living with HIV. Front Immunol. 2023;14:1173956. 37153621 10.3389/fimmu.2023.1173956 PMC10160480

[40] Rallón N García M García-Samaniego J. HCV coinfection contributes to HIV pathogenesis by increasing immune exhaustion in CD8 T-cells. PLoS One. 2017;12:e0173943. 28323897 10.1371/journal.pone.0173943 PMC5360268

[41] Catania A Manfredi MG Airaghi L. Plasma concentration of cytokine antagonists in patients with HIV infection. Neuroimmunomodulation. 1994;1:42–9. 8528884 10.1159/000097089

[42] Crisà E Boggione P Nicolosi M. Genetic predisposition to myelodysplastic syndromes: a challenge for adult hematologists. Int J Mol Sci . 2021;22:2525. 33802366 10.3390/ijms22052525 PMC7959319

[43] Dye CK Corley MJ Li D. Comparative DNA methylomic analyses reveal potential origins of novel epigenetic biomarkers of insulin resistance in monocytes from virally suppressed HIV-infected adults. Clin Epigenetics. 2019;11:1–9. 31253200 10.1186/s13148-019-0694-1 PMC6599380

[44] Fattizzo B Giannotta JA Barcellini W. Mesenchymal stem cells in aplastic anemia and myelodysplastic syndromes: the “seed and soil” crosstalk. Int J Mol Sci . 2020;21:5438. 32751628 10.3390/ijms21155438 PMC7432231

[45] Dharan NJ Yeh P Bloch M; ARCHIVE Study Group. HIV is associated with an increased risk of age-related clonal hematopoiesis among older adults. Nat Med. 2021;27:1006–11. 34099923 10.1038/s41591-021-01357-y

[46] Donnelly MR Ciborowski P. Proteomics, biomarkers, and HIV‐1: a current perspective. Proteomics Clin Appl. 2016;10:110–25. 26033875 10.1002/prca.201500002 PMC4666820

[47] Patters BJ Kumar S. The role of exosomal transport of viral agents in persistent HIV pathogenesis. Retrovirology. 2018;15:79. 30577804 10.1186/s12977-018-0462-x PMC6303896

[48] Borjabad A Brooks AI Volsky DJ. Gene expression profiles of HIV-1-infected glia and brain: toward better understanding of the role of astrocytes in HIV-1-associated neurocognitive disorders. J Neuroimmune Pharmacol. 2010;5:44–62. 19697136 10.1007/s11481-009-9167-1 PMC3107560

[49] Liberal R Mieli-Vergani G Vergani D. Clinical significance of autoantibodies in autoimmune hepatitis. J Autoimmun. 2013;46:17–24. 24016388 10.1016/j.jaut.2013.08.001

[50] Votavova H Belickova M. Hypoplastic myelodysplastic syndrome and acquired aplastic anemia: immune-mediated bone marrow failure syndromes. Int J Oncol. 2021;60:7. 34958107 10.3892/ijo.2021.5297 PMC8727136

[51] Malone ER Oliva M Sabatini PJB Stockley TL Siu LL. Molecular profiling for precision cancer therapies. Genome Med. 2020;12:8. 31937368 10.1186/s13073-019-0703-1 PMC6961404

[52] Lee EY Kulkarni RP. Circulating biomarkers predictive of tumor response to cancer immunotherapy. Expert Rev Mol Diagn. 2019;19:895–904. 31469965 10.1080/14737159.2019.1659728 PMC6773262

[53] Narita A Kojima S. Biomarkers for predicting clinical response to immunosuppressive therapy in aplastic anemia. Int J Hematol. 2016;104:153–8. 27091471 10.1007/s12185-016-2009-z

